# Myxoid liposarcoma with negative features on bone scan and [18F]-2-fluoro-2-deoxy-D-glucose-positron emission tomography

**DOI:** 10.1186/1477-7819-10-214

**Published:** 2012-10-09

**Authors:** Akio Sakamoto, Yoshiaki Fukutoku, Yoshihiro Matsumoto, Katsumi Harimaya, Yoshinao Oda, Yukihide Iwamoto

**Affiliations:** 1 Department of Orthopaedic Surgery, National Hospital Organization Kokura Medical Center, Kitakyushu, Fukuoka 802-0803, Japan; 2 Department of Orthopaedic Surgery, Graduate School of Medical Sciences, Kyushu University, Fukuoka, 812-8582, Japan; 3 Department of Anatomic Pathology, Graduate School of Medical Sciences, Kyushu University, Fukuoka, 812-8582, Japan

**Keywords:** Myxoid liposarcoma, Bone scan, FDG-PET, Vertebral metastasis

## Abstract

**Background:**

Myxoid liposarcoma occurs in middle age, and is characterized by extrapulmonary metastasis, including bone metastasis. Bone scans and [18F]-2-fluoro-2-deoxy-D-glucose-positron emission tomography (FDG-PET) are widely used for assessment of tumor extension, including vertebral metastasis. However, both methods have a low positive rate with regard to vertebral metastasis arising from myxoid liposarcoma. This is particularly true for bone scans for intramedullary lesions that have no cortical involvement.

**Case presentation:**

We present the case of a 53-year-old male with myxoid liposarcoma in the leg. He had been treated for multiple metastases over a ten-year period, and was experiencing back pain due to a pathological fracture in the second lumbar vertebra (L2). Magnetic resonance imaging of all the vertebrae showed abnormal signal intensity suggestive of metastasis in eight vertebrae, and revealed extraskeletal extension in three vertebrae. Bone scans and FDG-PET were negative except for the L2 fracture which was indicated on a bone scan.

**Conclusions:**

Both bone scans and FDG-PET can be negative in cases of vertebral metastasis that arise from myxoid liposarcoma, even when extraskeletal extensions are present. Similarly, even a fractured vertebra may not always be visible on FDG-PET.

## Background

Myxoid liposarcoma is one of the subtypes of liposarcoma. This subtype typically peaks in the fourth and fifth decades of life [1]. Myxoid liposarcoma is commonly associated with TLS-CHOP fusion transcript [2,3]. In contrast to other soft-tissue sarcomas that show a tendency for metastasis to the lung, myxoid liposarcoma has a propensity to spread to extrapulmonary sites, including bone [4,5]. The frequency of bone metastasis arising from myxoid liposarcoma has been reported to be 14% [1] and 17% [6].

Bone scans and [18F]-2-fluoro-2-deoxy-D-glucose-positron emission tomography (FDG-PET) are useful for detecting distant metastases from various tumors. Bone scans are sensitive to unusual bone remodeling activity because radioactive material is taken up by osteoblasts. On the other hand, FDG-PET can be evaluated using the standardized uptake value (SUV). An increased SUV reflects increased glucose metabolism as a result of various factors such as increased glucose transporters, high levels of hexokinase and a reduction in glucose-6-phosphatase [7,8]. FDG-PET has been reported to be useful for the diagnosis of soft-tissue tumors [9].

Bone scans and FDG-PET have been reported to lack sufficient sensitivity for the detection of vertebral metastases arising from myxoid liposarcoma. Among vertebral metastases detected by magnetic resonance imaging (MRI), positive rates with bone scans and FDG-PET have been reported to be 16% and 14%, respectively, in a number of vertebrae [6]. It also has been reported that the positive rate with bone scans for vertebral metastasis of various tumors is rather low for intramedullary lesions that have no cortical involvement [10]. In the current study, we report the case of a man with myxoid liposarcoma with multiple vertebral metastases. Although some of the metastatic vertebrae had extended extraskeletally, all the metastatic vertebral lesions were negative on bone scans and FDG-PET, except for the fractured vertebra, which was visible on a bone scan.

## Case presentation

**Figure 1:**
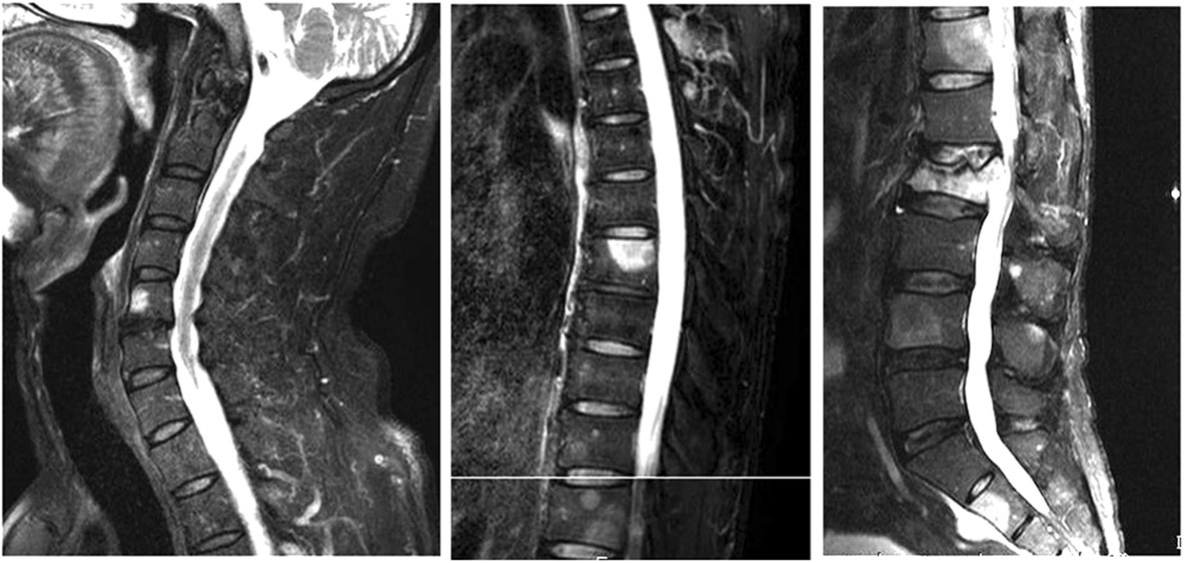
**Magnetic resonance imaging shows high signal intensity on a T2-image with fat suppression in multiple vertebrae.** Pathological fracture is evident in the second vertebra. Extraskeletal extension is visible in the eleventh thoracic vertebra, second lumbar vertebra and second sacral vertebra.

**Figure 2:**
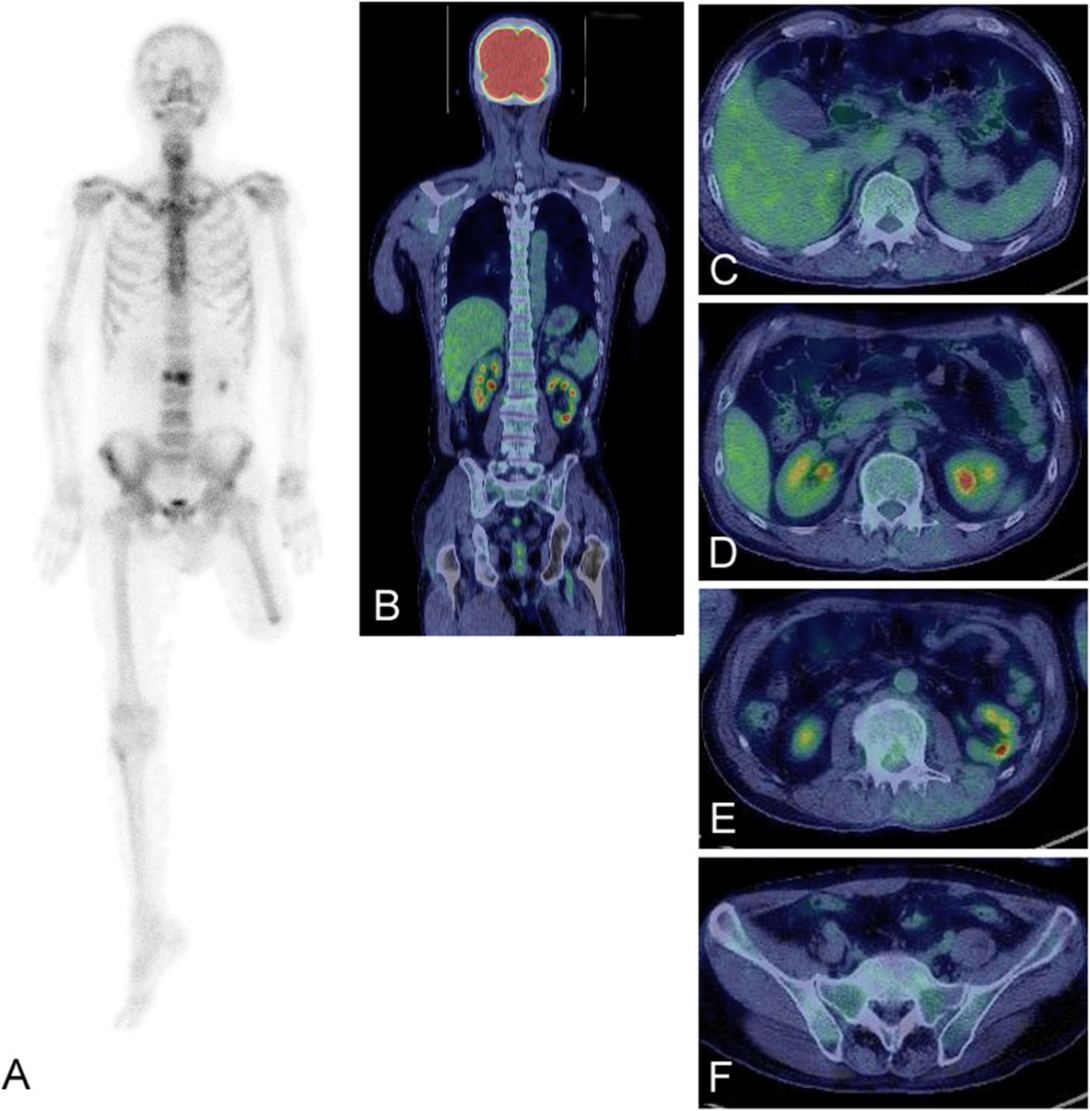
**Positive findings can be seen on the bone scan in the fractured second lumbar vertebra (L2)(A).** Coronal section (**B**) and axial sections of twelfth thoracic vertebra (T12) (**C**), L1 (**D**), L2 (**E**) and the first sacral vertebra (S1) (**F**) in FDG-PET are shown. There are no obvious differences between FDG uptake in metastatic vertebrae of T12 (**C**), L2 (**E**), and the non-metastatic vertebrae of L1 (**D**) and S1 (**F**). FDG-PET, [18F]-2-fluoro-2-deoxy-D-glucose-positron emission tomography.

A 43-year-old man with a tumor in his left leg was referred to our institute. The diagnosis was myxoid liposarcoma based upon biopsy findings. Radiation and chemotherapy were administered. Consequent wide resection with the surrounding normal tissue was performed. Two years after the initial surgery, when the patient was 45 years of age, metastasis appeared in the right neck region and the retroperitoneum, and resection of these lesions was performed. Seven years after the initial surgery, when the patient was 50 years of age, lung metastasis was found and resection was performed. Eight years after the initial surgery, when the patient was 51 years of age, recurrence was found in the thigh and involved the major vessels. Amputation at the thigh was carried out. Histological diagnosis of myxoid liposarcoma was confirmed in each of the resected materials. Chemotherapy was performed after every resection of metastatic and recurrent lesions. Ten years after the initial operation, at 53 years of age, the patient experienced back pain. Plain radiographs showed a compression type fracture in the second lumbar vertebra. MRI of all the vertebrae showed abnormally high signal intensity on T2-imaging with fat suppression in eight vertebrae: the fifth cervical vertebra (C5), the seventh, tenth, eleventh and twelfth thoracic vertebrae (T7, T10, T11 and T12), the second and fourth lumbar vertebrae (L2 and L4), and the second sacral vertebra (S2). High signal intensity on the MRI was seen throughout the entire vertebral body in L2 accompanied by deformity, suggestive of a pathological fracture. Extraskeletal extension of the lesion was seen in the T12, L2 and S2 vertebrae (Figure 1). Bone scans showed negative findings except for the L2 fracture. FDG-PET was negative for all of the vertebrae, including the fractured L2 vertebra (Figure 2). These examinations of bone scans and FDG-PET were performed within one month after the MRI examination. With a diagnosis of vertebral metastasis arising from myxoid liposarcoma, radiation and chemotherapy were administered. The patient was informed that data from his case would be submitted for publication, and his consent was obtained.

## Conclusions

Myxoid liposarcoma is characteristic in giving rise to extrapulmonary metastasis, such as to the retroperitoneum, the axilla and the mesentery, while other soft-tissue sarcomas demonstrate a tendency to metastasize to the lung [3,11,12]. The overall rate of metastasis in myxoid liposarcoma has been reported to be one-third [2,6]. The frequency of bone metastasis arising from myxoid liposarcoma has been reported to be 14% [1] and 17% [6]. The actual frequency of vertebral metastasis of myxoid liposarcoma is not clear, since a low metastatic rate of 4.3% has also been reported [13].

Bone scans are known to be more sensitive than plain radiographs for detecting bone metastasis [13]. A low positive rate with bone scans has been reported for vertebral metastasis arising from myxoid liposarcoma. In a previous report, bone scans revealed three out of nine cases (33%) and five out of 32 vertebrae (16%), based upon known metastasis as shown on MRI [6]. As for the sensitivity of bone scans, a lower positive rate for metastatic bone tumors in various malignancies has been reported in cases of intramedullary lesions without cortical involvement [10]. In the current report, although the vertebral lesions had extended extraskeletally in three vertebrae, bone scans of the vertebrae were negative in all cases except for the one vertebra that was fractured. The reason for this low positive rate in cases of myxoid liposarcoma is still not clear. In certain conditions such as myeloma or lesions confined to the marrow, bone scans have shown low sensitivity [14]. It has been suggested that hematogenously seeded intramedullary metastasis produces marrow replacement lesions without destroying the bone structure [15]. Moreover, myeloma cells promote osteoclast activity but inhibit osteoblast differentiation, which may explain the negativity of bone scans [16].

An increased uptake of FDG-PET in cells reflects increased glucose metabolism [7,8], and has been used to evaluate the extent of disease. As for bone and soft-tissue tumors, it has been reported that malignant tumors tend to have a higher SUV than benign tumors [9]. A low positive rate of FDG-PET has been reported in cases of vertebral metastasis of myxoid liposarcoma. In a previous report, FDG-PET revealed two out of six cases (33%), and four out of 29 vertebrae (14%), based upon known metastasis as shown on MRI [6]. The reason FDG-PET has a lower positive rate in cases of vertebral metastasis of myxoid liposarcoma is not clear. It has been assumed that the myxoid stroma in myxoid liposarcoma may prevent the labeled glucose from reaching cells in a sufficient quantity to be detected by the scanner [6]. Interestingly, in the current case, SUV was still negative in FDG-PET even in the fractured vertebra, whereas positive results were seen on the bone scan. A fracture site is usually positive on FDG-PET. The discrepancy between the results of the FDG-PET and the bone scan for the fractured vertebra could be associated with a specific biological mechanism, such as mild inhibition of the healing reaction at the fracture site.

In summary, a case of myxoid liposarcoma with multiple vertebral metastases is reported. Bone scans and FDG-PET were negative, even for lesions with extraskeletal extension, except for the L2 fracture which was visible on a bone scan. Bone scans and FDG-PET can thus be negative in cases of multiple vertebral metastases of myxoid liposarcoma, and even a fractured vertebra may not be visible on FDG-PET.

## Consent

Written informed consent was obtained from the patient for publication of this case report and any accompanying images. A copy of the written consent is available for review by the Editor-in-Chief of this journal.

## Abbreviations

FDG-PET: [18F]-2-fluoro-2-deoxy-D-glucose-positron emission tomography; MRI: magnetic resonance imaging; SUV: standardized uptake value; C: cervical vertebra; T: thoracic vertebra; L: lumbar vertebra; S: sacral vertebra.

## Competing interests

The authors declare that they have no competing interests.

## Authors’ contributions

AS drafted the manuscript. AS, YF, YM, KH and YI administered the treatment. OY and YI conceived the study, participated in its design and coordination, and helped to draft the manuscript. All authors read and approved the final manuscript.
